# Chemotherapy in Metastatic NSCLC – New Regimens (Pemetrexed, Nab-Paclitaxel)

**DOI:** 10.3389/fonc.2014.00177

**Published:** 2014-07-21

**Authors:** Normand Blais, Vera Hirsh

**Affiliations:** ^1^Centre hospitalier de l’Université de Montréal (CHUM), Hôpital Notre-Dame, Montreal, QC, Canada; ^2^McGill University Health Centre (MUHC), Royal Victoria Hospital, Montreal, QC, Canada

**Keywords:** metastatic, non-small cell lung carcinoma, solvent-based paclitaxel, nab-paclitaxel, pemetrexed, histology, clinical trials

## Abstract

Platinum-based chemotherapy doublets have been the standard approach to first-line therapy for more than a decade. Many randomized trials testing new combinations have not been able to produce significant gains in patient outcomes when these studies have looked at an unselected patient population. The recognition of the biologic importance of histology and molecular features of lung cancer has dramatically impacted on patient care, as can be easily recognized by the advent of targeted therapy for molecularly defined lung cancers. Similarly, for lung cancers without recognized driver mutations, subgroup evaluations of trials-based histology has identified that some chemotherapy regimens offer greater benefit in the squamous cell or the non-squamous cell groups. Two such examples are nab-paclitaxel and pemetrexed. These have shown improved anti-tumor activity and a decreased toxicity profile compared to standard combinations. Preferential activity in histologic divided patient subgroups can allow the clinician to personalize his approach to care. The role of these two agents in the management of NSCLC will be described in this article.

## Introduction

The standard of care for the first-line treatment of advanced NSCLC without epidermal growth factor receptor (EGFR) or anaplastic lymphoma kinase (ALK) mutations (about 80% of advanced NSCLC) remains a platinum-based doublet in patients with good performance status (PS) and no significant comorbidities. This includes third-generation cytotoxic agents (i.e., cisplatin plus gemcitabine or carboplatin plus paclitaxel) (1, 2). Until recently no platinum doublet has demonstrated superiority over another in the treatment of advanced NSCLC (3–10). Lately, histology has been shown to affect the treatment outcomes (1). Patients with non-squamous cell cancer (NSCC) currently have a variety of first-line treatment options (1). Testing for EGFR and ALK mutations and tailoring therapy accordingly are now accepted as a standard practice in patients with NSCLC (1). For these patients, current guidelines recommend targeted therapies as first-line treatment (1). However, about 60–90%, varying largely according to ethnicity and smoking status, of patients with NSCLC have wild-type EGFR. Several studies have demonstrated that EGFR mutations occur infrequently in patients with squamous cell carcinoma (11–14). ALK mutations/fusions have been observed in only 1–7% of patients (15–20). Because EGFR and ALK mutations occur infrequently in patients with squamous cell NSCLC, mutation testing is not recommended routinely with the exception of never smokers and mixed histologies (1, 21, 22). Overall, for patients with EGFR and Alk unmutated NSCLC, platinum-based chemotherapy regimens remain the standard of care in first-line therapy (1). Key phase III studies reporting outcomes with platinum-doublet regimens for patients with squamous histology have demonstrated a median overall survival (OS) ranging from 8.9 to 13.7 months (5, 23–26) whereas the NSCC population fared slightly better with an OS of 10.4–14.9 months. Similarly, recently reported trials have renewed the interest in adapting treatment based on histology. Two key examples of potential application of personalized therapy based on histology are nab-paclitaxel, which has shown improved results in the squamous cell cancer (SCC) population and pemetrexed, which has on the contrary, shown clear benefit in the NSCC patients. This mini-review will highlight these two unique drugs and how they can be incorporated into practice to improve outcomes in NSCLC therapy.

## Pemetrexed

Pemetrexed is a folic acid derivative that inhibits both purine and pyrimidine synthesis by blocking three key metabolic enzymes involved in DNA synthesis: thymidylate synthase (TS), dihydrofolate reductase (DHFR), and glycinamide ribonucleotide formyltransferase (GARFT) (27). Building on the efficacy of methotrexate in many human cancers, research focusing on the identification of more potent inhibitors of purine synthesis has led to the identification of the promising activity of pemetrexed in lung cancer in the late 1990s (27). Although several hundred trials have been conducted to evaluate the efficacy of pemetrexed in many cancer settings, the first pivotal study was reported by Hanna in 2004 (28). It is interesting to note that at this time in pemetrexed development, studies were conducted in unselected patient populations with NSCLC. Emerging science since 2003 (29) and in particular the study published by Ceppi et al. (30) in 2006, identified that chemonaïve patients with squamous carcinoma had tumors expressing higher levels of TS than patients with adenocarcinoma. These seminal studies led to the re-evaluation of previously published as well as ongoing randomized trials to evaluate the interaction of histology with clinical efficacy in NSCLC. The demonstration of a consistent effect in NSCC and the lack of effect in SCC (Table 1) has eventually directed the registration process for pemetrexed use and dictated its current clinical use, which is now restricted to non-squamous histology. Interestingly, knowledge of anti-folate metabolism has also been able to decrease the incidence of pemetrexed toxicity by the addition of folic acid and vitamin B12 supplementation (31).

**Table 1:** **Pemetrexed-histology interaction in randomized clinical trials of NSCLC**.

	First-line therapy (*n* = 1725)		Maintenance therapy (*N* = 663)		Second-line therapy (*N* = 571)	
	Squamous (*N* = 473)	Non-squamous (*N* = 1252)	Squamous (*N* = 182)	Non-squamous (*N* = 481)	Squamous (*N* = 172)	Non-squamous (*N* = 399)
**Overall survival**
Adjusted HR	1.23	0.84	1.07	0.07	1.56	0.78
Superiority *p*	0.050	0.011	0.678	0.002	0.018	0.048
Interaction test *p*	0.002		0.033		0.001	
**Progression-free survival**
Adjusted HR	1.36	0.95	1.03	0.47	1.40	0.82
Superiority *p*	0.002	0.349	0.896	<0.001	0.046	0.076
Interaction test *p*	0.002		0.036		0.004	

*Adapted from Scagliotti et al. (32)*.

*Hazard ratio <1 favors pemetrexed in non-squamous subgroups of all three studies*.

*Hazard ratio (HR)> 1.0 favors comparator arm in squamous subgroups of all three studies*.

## Second-Line Studies

Initially published in 2004 (28) as a study showing similar efficacy between pemetrexed and docetaxel in unselected patients with NSCLC, Hanna et al. presented a subgroup analysis based on histology at the 12th WCLC in Seoul, Korea in 2007. The reported median OS in the NSCC population was 9.2 vs. 8.2 months (pemetrexed vs. docetaxel) and this was respectively 6.2 vs. 7.4 months in the SCC cohort. This translates to an adjusted HR for OS of 0.78 in favor of pemetrexed in the NSCC group and a HR of 1.56 in the SCC group. Results were similar for PFS, with the HR being 0.82 in the NSCC group and 1.40 in the SCC group. The statistical test demonstrating a quantitative interaction was positive for OS (*p* = 0.001) and PFS (*p* = 0.004) (32).

## Maintenance Studies

Three large randomized, placebo-controlled trials address the issue of pemetrexed maintenance and are discussed in the article of this issue of Frontiers related to maintenance therapy. As the PARAMOUNT (33) and AVAPERL (34) study are composed of an exclusively NSCC population, only JMEN is informative as to the histological interaction of pemetrexed therapy in this setting. The JMEN study, published in 2009 by Ciuleanu (35) was reported as a positive trial in an unselected population. The PFS after first-line therapy was 4.3 months in the pemetrexed group and 2.6 months in the placebo group (HR 0.50, *p* < 0.001). The corresponding OS values were 13.4 and 10.6 months (HR 0.79, *p* = 0.012). To validate, the biologic relevance of the histologic interaction previously observed in the Hanna study, Belani et al. reported this subgroup analysis at ASCO in 2009. This analysis convincingly supported the previously observed finding of a histological effect. Whereas PFS was indeed favorably impacted in the NSCC population (HR 0.47, *p* < 0.0001), this was not the case for SCC (HR 1.03, *p* = 0.90). Similar findings were found for OS (HR 0.70, *p* = 0.002 and HR 1.07, *p* = 0.68). From this moment on, pemetrexed use in the setting of SCC decreased substantially. The histologic effect of pemetrexed was major contributors to the major revolution in lung cancer care. A diagnosis of NSCLC (non-otherwise specified) is now considered suboptimal for patient care.

## First-Line Studies

The JMDB study compared pemetrexed plus cisplatin with gemcitabine plus cisplatin in patients with advanced NSCLC (5). Similarly with other previously conducted trials evaluating pemetrexed, patients of all histologies were accrued to this trial that was published before the widespread acceptance of a clear histologic effect. Whereas, the overall study results did not appear to favor the pemetrexed plus cisplatin combination (OS HR 0.94, *p* = NS), subgroup analysis based on histology did show once again a significant interaction. The gemcitabine regimen produced a significantly longer median OS compared with pemetrexed regimen in patients with SCC (10.8 vs. 9.4 months, HR 1.23, *p* = 0.05). The opposite was seen in patients with NSCC (10.4 vs. 11.8 months, HR 0.84, *p* = 0.011). Similarly, PFS was impacted in a similar manner in the SCC group (HR 1.36, *p* = 0.002) and the NSCC group (HR 0.95, *p* = 0.35). The treatment-by-histology interaction test was positive for PFS and for OS.

One outlier in the pemetrexed–histology interaction story is the Gronberg trial published in 2009 (36). This smaller study included 436 patients, including 248 patients with NSCC histology. Median OS was 7.3 months in the pemetrexed with carboplatin arm compared to 7.0 months in the gemcitabine with carboplatin arm (*p* = 0.63). Subgroup analysis was respectively 7.8 vs. 7.5 in patients with NSCC (*p* = 0.77) and not reported for the SCC subgroup. This trial was not designed to evaluate RR or PFS as radiological assessment was not mandatory. These seemingly inferior results may be due to the patient population, which included 22% of patients with PS 2 status, to the upfront 25% decrease in pemetrexed dose in the 18% of the population that was >75 years of age, to the use of carboplatin instead of cisplatin, or to the small sample size of this study.

Although the combination of carboplatin with pemetrexed and bevacizumab has never been formally compared with standard cisplatin and pemetrexed, the PointBreak (37) study compared another standard regimen – carboplatin plus paclitaxel and bevacizumab compared with carboplatin with pemetrexed and bevacizumab. This extensive, 939-patient, exclusively-NSCC trial failed to show a clearly superior regimen in terms of efficacy. The HR for OS was 1.00 and for PFS was 0.83 (*p* = 0.012) slightly favoring the pemetrexed triplet. The contribution of bevacizumab to a platinum-pemetrexed doublet has never been studied in a randomized trial.

## Safety and Tolerability

The study by Hanna et al. (28) provides a good opportunity to compare pemetrexed and docetaxel’s toxicity profile. In this study, Grade 3–4 hematologic toxicities were significantly worse with docetaxel: neutropenia (40.2 vs. 5.3%), febrile neutropenia (12.7 vs. 1.9%), compared to pemetrexed.

Non-hematologic toxicities (all grades) were relatively similar except for alopecia (37.7 vs. 6.4%) and diarrhea (24.3 vs. 12.8%) more frequently observed with docetaxel and ALT elevations (1.4 vs. 7.9%) more frequent with pemetrexed.

Relevant to second-line therapy decisions, there have been no large randomized trial comparing pemetrexed alone and an EGFR inhibitor in the second-line setting. A small phase II randomized trial did compare pemetrexed to erlotinib in EGFR mutation-negative but EGFR–FISH positive NSCLC (38). Although efficacy differences could not be demonstrated between both regimens, toxicity analysis did provide interesting observations. Few Grade 3–4 toxicities were described. For erlotinib and pemetrexed, respectively, rash was present in 3.3 and 0%; diarrhea in 1.6 and 0%; anorexia in 1.6 and 0%, and nausea in 0 and 3.2% (38). Compared to placebo in the Ciuleanu maintenance trial, patients having received four courses of a first-line platinum containing doublet rarely developed Grade 3–4 toxicities (all <5%) while on pemetrexed alone. The most frequent toxicities (mostly Grade 1–2) were fatigue (24 vs. 10%), anorexia (19 vs. 2%), and nausea (19 vs. 5%); all compared to placebo.

The first difference between cisplatin with pemetrexed and cisplatin with gemcitabine is its administration schedule in that the latter regimen requires a second visit to the chemotherapy suite on day 8 whereas the former is a day 1 infusion alone. The JMDB trial also showed some differences in the Grade 3–4 toxicity profile of both combinations. Compared with the pemetrexed regimen, the gemcitabine doublet was associated with more neutropenia (26.7 vs. 15.1%), febrile neutropenia (3.7 vs. 1.3%), anemia (7.6 vs. 4.8%) and thrombocytopenia (12.7 vs. 4.1%), and less anorexia (0.7 vs. 2.4%) and nausea (3.9 vs. 7.2%).

In the Gronberg trial, the use of pemetrexed and carboplatin was associated with less granulocytopenia (40 vs. 51%) and thrombocytopenia (24 vs. 56%) compared to carboplatin and gemcitabine. This was associated with a decrease in the transfusion of red blood cell (29 vs. 43%) and platelet (3 vs. 9%) transfusions with pemetrexed use (5).

The PointBreak trial showed clinically relevant differences in the Grade 3–4 toxicities of the studied regimens. The pemetrexed arm was associated with more anemia (14.5 vs. 2.7%), thrombocytopenia (23.3 vs. 5.6%), and fatigue (10.9 vs. 5.0%) whereas neutropenia (40.6 vs. 25.8%), febrile neutropenia (4.1 vs. 1.4%), sensory neuropathy (4.1 vs. 0%), and alopecia (36.8 vs. 6.6%) occurred more frequently in the paclitaxel triplet (37).

## Conclusion

Pemetrexed use has shown consistent effects in favor of its use in the setting of non-squamous cell lung cancer. Compared with paclitaxel, gemcitabine, or docetaxel, its favorable efficacy, toxicity profile, and convenient schedule of administration makes this an agent of choice in this setting. As discussed in the article of this journal on maintenance therapy, data from the PARAMOUNT and AVAPERL trial lends increasing support for the first-line use of pemetrexed and its consideration for maintenance in non-progressing patients after induction therapy.

## Nab-Paclitaxel

Nab-paclitaxel is a 130 nm, albumin-bound formulation of the microtubule inhibitor paclitaxel (39), and is a solvent-free option for the first-line treatment of advanced NSCLC (39).

Solvents such as Cremophor EL and polysorbate 80 require specialized tubing for administration, which is not required for the administration of nab-paclitaxel (39–42). These solvents have intrinsic toxicities, i.e., hypersensitivity reactions requiring steroid/antihistamine pre-treatments, neuropathy, and excessive fluid retention (40, 43, 44). In a pre-clinical study, nab-paclitaxel demonstrated both enhanced endothelial cell binding and transport, and improved delivery of paclitaxel to tumors compared with solvent-based paclitaxel (45).

A phase III trial demonstrated overall response rates (ORRs) superior for nab-paclitaxel plus carboplatin compared with conventional solvent-based paclitaxel plus carboplatin in patients with advanced NSCLC (46). Patient populations, i.e., with squamous histology and those ≥70 years of age, had improved clinical outcomes with nab-paclitaxel plus carboplatin compared with solvent-based paclitaxel plus carboplatin (47, 48). No unexpected differences in toxicity were noted.

## Clinical Efficacy

In the multicenter, randomized, Phase III registrational trial, 1052 patients with advanced NSCLC were randomized to receive first-line weekly nab-paclitaxel (100 mg/m^2^) plus carboplatin (AUC 6) every 3 weeks (*n* = 521) or solvent-based (sb) paclitaxel (200 mg/m^2^) plus carboplatin (AUC 6) every 3 weeks (*n* = 531) (46). Patients had to have stage IIIB/IV disease, ECOG PS of 0 or 1 and were previously untreated for metastatic NSCLC. Adjuvant chemotherapy was permitted if it was completed 12 months prior to study enrollment. The median age of the patients was 60 years, 75% were male, 81% were white, 73% were smokers, and 79% had stage IV disease. Patients in the nab-paclitaxel arm had a significantly greater ORR = the primary endpoint, compared with the sb-paclitaxel arm (33 vs. 25%, response rate ratio = 1.313, 95% CI = 1.082–1.593, *p* = 0.005). Patients on nab-paclitaxel had longer median PFS = 6.3 vs. 5.8 months, hazard ratio (HR) = 0.902, 95% CI = 0.767–1.060, *p* = 0.214). The median OS for nab-paclitaxel vs. sb-paclitaxel was 12.1 vs. 11.2 months (HR = 0.922, 95% CI = 0.797–1.066, *p* = 0.271), respectively.

An exploratory elderly subgroup analysis examined the efficacy and safety of these two regimens in patients ≥70 years of age enrolled in this phase III trial (46). In these patients (*n* = 156), the ORR in the nab-paclitaxel vs. sb-paclitaxel arms was 34 vs. 24% (*p* = 0.196). A trend toward improved PFS was also noted in elderly patients with nab-paclitaxel, 8.0 vs. 6.8 months, *p* = 0.134. Elderly patients in the nab-paclitaxel arm experienced an impressive 19.9 months median OS compared with 10.4 months in the sb-paclitaxel arm, *p* = 0.009.

Another analysis of this phase III trial examined efficacy of the regimens by histology (48). Patients with squamous NSCLC (*n* = 450), achieved a significantly higher ORR, *p* < 0.001, with nab-paclitaxel plus carboplatin (41%) vs. sb-paclitaxel plus carboplatin (24%) and a 1.2 month improvement in median OS (10.7 vs. 9.5 months, *p* = NS). A similar ORR was observed for nab-paclitaxel plus carboplatin vs. sb-paclitaxel in patients with non-squamous NSCLC (*n* = 602, 26 vs. 25%, *p* = NS), median OS was 13.1 vs. 13 months in each arm (*p* = NS). Table 2 shows efficacy outcomes of the intent-to-treat (ITT) population as well as by age and histology from the phase III trial of nab-paclitaxel plus carboplatin in NSCLC.

**Table 2:** **Select efficacy outcomes from the Phase III trial of nab-paclitaxel plus carboplatin in NSCLC**.

Treatment	ITT (41)		≥70 years (42)		Histology (43)			
					SCC		NSCC	
	nab-P/C	sb-P/C	nab-P/C	sb-P/C	nab-P/C	sb-P/C	nab-P/C	sb-P/C
*n*	514	524	74	82	229	221	292	310
ORR (%)	33	25	34	24	41	24	26	25
Response rate ratio	1.313		1.385		1.680		1.034	
*p*-Value	0.005		0.196		<0.001		0.808	
Median PFS (months)	6.3	5.8	8.0	6.8	5.6	5.7	6.9	6.5
HR	0.902		0.687		0.865		0.933	
*p*-Value	0.214		0.134		0.245		0.532	
Median OS (months)	12.1	11.2	8.0	6.8	10.7	9.5	13.1	13.0
HR	0.922		0.583		0.890		0.950	
*p*-Value	0.271		0.009		0.284		0.611	

*HR, hazard ratio; IIT, intent-to-treat; nab-P/C, nab-paclitaxel + carboplatin; NSCC, non-squamous cell carcinoma; NSCLC, non-small cell lung cancer; ORR, overall response rate; OS, overall survival; PFS, progression-free survival; sb-P/C, solvent-based paclitaxel + carboplatin; SCC, squamous cell carcinoma*.

## Safety and Tolerability

The most common Grade 3–4 adverse events of the ITT population and select subgroups of the phase III trial are shown in Table S1 in Supplementary Material.

Pre-treatment with antihistamines and/or steroids is required for sb-paclitaxel and docetaxel to prevent hypersensitivity reactions but not for nab-paclitaxel (39, 42). Much of these reactions may be solvent-related because both Cremophor EL and polysorbate 80 have been associated with hypersensitivity reactions (40); nab-paclitaxel is not formulated with a chemical solvent (39). Taxanes are associated with the development of peripheral neuropathy (49). However, in the phase III trial of nab-paclitaxel plus carboplatin vs. sb-paclitaxel plus carboplatin, patients on nab-paclitaxel arm experienced significantly less grade 3–4 peripheral neuropathy compared with the sb-paclitaxel arm (46). Results based on the FACT-Taxane neuropathy, pain in hands/feet and hearing loss subscales demonstrated significantly less worsening of taxane-related symptoms in the nab-paclitaxel arm compared with the sb-paclitaxel arm, *p* ≤ 0.002 for all (46, 50). The patient-reported symptom scores were consistent with physician assessments of peripheral neuropathy (50). In addition, patients in the nab-paclitaxel arm who experienced Grade 3–4 peripheral neuropathy experienced a faster median time-to-improvement to Grade 1 (38 vs. 104 days) compared to sb-paclitaxel, respectively.

Taxane use is frequently associated with increased muscle and joint pains (39, 42). In the phase III study, patients in the nab-paclitaxel arm experienced significantly less Grade 3–4 arthralgia and myalgia than patients in the sb-paclitaxel arm (46, 50). In the phase III trial, patients in the nab-paclitaxel arm experienced significantly less Grade 3–4 neutropenia, but *more* thrombocytopenia and anemia *than* patients in the sb-based arm (46, 50).

## Conclusion

Nab-paclitaxel represents an important advancement especially as the treatment options for patients with squamous histology are limited and elderly patients are often undertreated due to toxicity concerns among other reasons. Based on these findings and its greater ease of administration, nab-paclitaxel plus carboplatin could be considered a first-line standard of care therapy in patients with advanced NSCLC. Targeted agents active for patients with squamous histology are in development and in the near future some of these agents could be assessed in combination with this regimen.

We must also select for each patient a treatment that is best suited to his individual comorbidities and treatment toxicities to ensure the best possible QOL during the last months of his life. Better safety and tolerability profile in addition to a greater RR makes nab-paclitaxel an excellent improvement to a paclitaxel combination for the first-line treatment of metastatic NSCLC especially for squamous cell carcinoma.

## Conflict of Interest Statement

The authors declare that the research was conducted in the absence of any commercial or financial relationships that could be construed as a potential conflict of interest.

## Supplementary Material

The Supplementary Material for this article can be found online at http://www.frontiersin.org/Journal/10.3389/fonc.2014.00177/abstract
