# Erratum: Chemotherapy in Metastatic NSCLC – New Regimens (Pemetrexed, Nab-Paclitaxel)

**DOI:** 10.3389/fonc.2014.00300

**Published:** 2014-10-30

**Authors:** Frontiers Production Office

**Affiliations:** ^1^Frontiers Production Office, Frontiers, Switzerland

**Keywords:** metastatic, non-small cell lung carcinoma, solvent-based paclitaxel, nab-paclitaxel, pemetrexed, histology, clinical trials

## Reason for Erratum

In Table 2, the Median OS values (in months) in the column ≥70 years were changed due to a typesetting error. This error does not change the scientific conclusions of the article in any way. The publisher apologizes for this error and the correct version of Table 2 appears below.

**Table 2:** **Select efficacy outcomes from the Phase III trial of nab-paclitaxel plus carboplatin in NSCLC**.

Treatment	ITT (41)			≥70 years (42)			Histology (43)					
							SCC			NSCC		
	nab-P/C		sb-P/C	nab-P/C		sb-P/C	nab-P/C		sb-P/C	nab-P/C		sb-P/C
*n*	514		524	74		82	229		221	292		310
ORR (%)	33		25	34		24	41		24	26		25
Response rate ratio		1.313			1.385			1.680			1.034	
*p*-Value		0.005			0.196			<0.001			0.808	
Median PFS (months)	6.3		5.8	8.0		6.8	5.6		5.7	6.9		6.5
HR		0.902			0.687			0.865			0.933	
*p*-Value		0.214			0.134			0.245			0.532	
Median OS (months)	12.1		11.2	19.9		10.4	10.7		9.5	13.1		13.0
HR		0.922			0.583			0.890			0.950	
*p*-Value		0.271			0.009			0.284			0.611	

*HR, hazard ratio; IIT, intent-to-treat; nab-P/C, nab-paclitaxel + carboplatin; NSCC, non-squamous cell carcinoma; NSCLC, non-small cell lung cancer; ORR, overall response rate; OS, overall survival; PFS, progression-free survival; sb-P/C, solvent-based paclitaxel + carboplatin; SCC, squamous cell carcinoma*.

