# Supplementary material for: Chemotherapy in Metastatic NSCLC – New Regimens (Pemetrexed, Nab-Paclitaxel)
Source: Front Oncol. 2014 Jul 21;4:177. doi: 10.3389/fonc.2014.00177 (PMC4104641; doi:10.3389/fonc.2014.00177)
Supplement: Supplementary file 1 [file Table_1.PDF]

Table S1 | Select Grade 3–4 adverse events in the Phase III trial of nab-paclitaxel plus carboplatin vs. solvent-based paclitaxel plus carboplatin in NSCLC.

Treatment	ITT (41)		≥70 years (42)		Histology (43)			
					SCC		NSCC	
	nab-P/C	sb-P/C	nab-P/C	sb-P/C	nab-P/C	sb-P/C	nab-P/C	sb-P/C
**HEMATOLOGIC ABNORMALITIES**								
n	514	524	73	81	222	214	286	299
Anemia (%)	27	7[a]	23	10[a]	27	4[a]	28	9[a,b]
Neutropenia (%)	47[c]	58	54[c]	74	43	51	50[c]	63
Thrombocytopenia (%)	18	9[a]	23	14	21	7[a]	16	11
**NON-HEMATOLOGIC EVENTS**								
n	514	524	73	81	226	218	288	306
Sensory neuropathy (%)	3[c]	12	7[c]	23	3[c]	11	3[c]	12
Arthralgia (%)	0	2	0	2	0[c]	<1	0[c]	2
Myalgia (%)	<1	2	0	1[c]	<1	<1	0[c]	3

IIT, intent-to-treat; nab-P/C, nab-paclitaxel + carboplatin; NSCC, non-squamous cell carcinoma; NSCLC, non-small cell lung cancer; ORR, overall response rate; OS, overall survival; PFS, progression-free survival; sb-P/C, solvent-based paclitaxel + carboplatin; SCC, squamous cell carcinoma.

[a] *$p < 0.05$ in favor of sb-P/C.*

[b] *$n = 300$.*

[c] *$p < 0.05$ in favor of nab-P/C.*
